# Green infrastructure can limit but not solve air pollution injustice

**DOI:** 10.1038/s41467-021-24892-1

**Published:** 2021-08-03

**Authors:** Viniece Jennings, Colleen E. Reid, Christina H. Fuller

**Affiliations:** 1 Department of Public Health, Agnes Scott College, Decatur, GA USA; 2 Geography Department, University of Colorado Boulder, Boulder, CO USA; 3 Department of Population Health Sciences, Georgia State University School of Public Health, Atlanta, GA USA

**Keywords:** Environmental impact, Environmental impact

## Abstract

Outdoor air pollution contributes to millions of deaths worldwide yet air pollution has differential exposures across racial/ethnic groups and socioeconomic status. While green infrastructure has the potential to decrease air pollution and provide other benefits to human health, vegetation alone cannot resolve health disparities related to air pollution injustice. We discuss how unequal access to green infrastructure can limit air quality improvements for marginalized communities and provide strategies to move forward.

Outdoor air pollution is a leading contributor to the environmental burden of disease and linked to over four million deaths worldwide each year^1^. The World Health Organization (WHO) reports that almost half of cities with more than 100,000 residents, and most (97%) cities in low- and middle-income countries of that size, do not meet WHO air quality guidelines^2^. From 1960 to 2009, global levels of fine particulate matter increased by 38% leading to a greater health burden from polluted air^3^. Even in the midst of the current pandemic, evidence of higher COVID-19 deaths among people with pre-existing conditions was linked to elevated air pollution exposure^4,5^ and/or residing in areas with historically higher levels of air pollution^6^. Exposure to air pollution, however, is not evenly distributed, especially within cities. Many studies document differential exposure to air pollution by race/ethnicity^7,8^ and socio-economic status^8–10^ in cities across the world. Neighborhoods segregated by race and class often have less political and economic power, and are often neglected by government institutions such that they receive fewer resources compared to privileged communities^11^. This predicament results in disproportionate and overlapping exposures to environmental burdens^11^. Through the years, some approaches to decrease outdoor pollution include regulation of air pollution sources, emission controls on personal vehicles, and—more recently—the expansion of green infrastructure. The expansion of green infrastructure can mitigate urban air pollution to some extent. Yet, urban greening cannot compensate for systemic injustices that lead to disproportionate burdens in environmental health, and therefore, green infrastructure investments need to be balanced with other efforts to ameliorate air pollution injustices.

## Green infrastructure for air pollution mitigation

Many cities have explored the potential of green infrastructure to mitigate urban air pollution and studies estimate that the value of this ecosystem service is worth about $3.8 billion dollars in the United States alone^12^. The definition of green infrastructure varies by discipline and scope. In this article, we focus on the broader role of all urban vegetation that provides ecosystem services via the mitigation of air pollution. For example, roadside green barriers can block the movement of traffic pollution into surrounding communities, mitigating some air pollution exposure and its resulting negative health effects^13^.

Green infrastructure is one variable that makes up the collective infrastructure that supports city dwellers. While the extent of air pollution removal by green infrastructure can vary^14^, many researchers note the benefits of green infrastructure to a city’s ambient environment^12,15^. In some cases, increased tree density and leaf area index are associated with a variety of health benefits including fewer cases of respiratory illness^16^. The net benefits of green infrastructure, however, should be balanced with their potential to increase pollen and other compounds that contribute to air pollution^17^. Special attention to tree size, condition, density, and species is also needed to increase a tree’s capacity to provide benefits and decrease disservices^14^. Although green infrastructure can provide benefits to urban air, urban settings have their own stressors (e.g., compaction, high levels of air pollution) that can impede the ability of green infrastructure to provide ecosystem services. For example, factors such as meteorology, mixture of air pollution, and urban layout can affect the ability of green infrastructure to remove air pollution^18^. Along with ecosystem services related to air pollution removal, mounting studies document various physical and mental health benefits related to green spaces^19–23^. Green infrastructure in the form of parks, street trees, and other urban vegetation can also buffer against health disparities for conditions such as obesity, cardiovascular disease, psychological distress, and heat-related illness^24^.

## Unequal access to green infrastructure

While there is ample evidence for health benefits of vegetation, widespread inequities in urban vegetation by race and income^25,26^ prompt concerns of limited ecosystem services from green infrastructure in marginalized communities around the world. For example, findings of disparate access to green infrastructure are documented in parts of Canada^27^, South Africa^28,29^, the United Kingdom^30^, China^31^, and Colombia^32^. Many underlying drivers (e.g., exclusionary practices) that result in unequal exposure to environmental burdens reflect factors that also lead to unequal access to green infrastructure^33^. Systemic racism prompted practices such as residential segregation in various locations^34^. Residential segregation can force racial and ethnic populations to be located in areas with limited resources, greater community stressors, and exposure to pollutants that contribute to environmental health disparities^35^. To illustrate, the report ‘Toxic Waste and Race at Twenty,’ found that race continues to be the most important factor determining hazardous waste facility siting in the U.S^36^. Also, scholars describe how the legacy of apartheid and segregation negatively influenced the availability and access to urban green infrastructure in South Africa today^29^. Another example of this was redlining—a discriminatory mortgage appraisal process that began in the 1930s in the U.S^36^—which has been linked to disparate air pollution exposures^34^, health disparities^37^, and inequitable access to green infrastructure^36,38^. For example, Schell et al.^34^ describes how redlined neighborhoods have on average twenty-one percent less tree canopy compared to other communities. A study in Baltimore, Maryland observed that patterns of residential segregation contributed to the unequal distribution of green infrastructure and greater presence of stressors such as pollution, flooding, and urban heat islands^39^. Variations of green infrastructure access can also relate to the type of vegetation, urban form, and methodological approach being explored^30^. Therefore, unequal access to green infrastructure and its ecosystem services can have various implications in environmental health.

## Part of the solution, but not a panacea

Given the range of benefits from green infrastructure, more attention should be directed to sustainably increase the presence of and access to it within vulnerable urban communities. We acknowledge, however, that the strategy to reduce air pollution’s inequitable health impacts should not rest solely on the effectiveness of green infrastructure. Multiple systematic and long-lasting processes of discrimination (e.g., inequitable citing of industrial facilities and high traffic roads^33–38^) have resulted in unequal exposure to air pollution. Therefore, one strategy alone cannot solve the associated problems of inequitable exposure to air pollution. Without addressing these persistent and structural factors, green infrastructure can only taper air pollution injustice, without solving it sustainably.

Similar to other environmental perils, air pollution in disadvantaged communities must be mitigated at the source—through regulating pollution emissions equitably and dismantling systems that lead to disproportionate exposures in the first place. Improving management strategies also applies to other sectors of the environmental field. Comparable to the sentiment expressed in Hardin’s Tragedy of the Commons^37^, underestimating the importance of sustainable stewardship of natural resources will lead to environmental and health burdens. The idea that the social forces that lead to inequitable systems should then govern and allocate the benefits that nature offers perpetuates environmental injustice. For example, developing cities in a way that regards vegetation as merely an aesthetic accessory instead of a key part of its ecological backbone can be detrimental in many ways. On the other hand, increasing natural amenities in disadvantaged communities can result in gentrification if housing protections are not put into place^38^. Solutions are needed that relieve the burden that air pollution has on public health through green infrastructure while not inducing further harm to environmental justice communities. A key component of this is the comprehensive inclusion of affected communities at all levels of decision-making.

## Making green infrastructure work to promote air pollution justice

Given these systemic issues, we suggest that green infrastructure development be partnered with actions to ensure equity and environmental justice. First of all, environmental justice calls for the involvement of diverse residents in environmental decision making. With concerns that urban green space development may lead to population displacement (i.e., green gentrification)^39,40^ and strains to public health^41^, re-engaging community members and professionals in this arena is imperative. Surface level involvement of local communities is not sufficient to mitigate such effects^25^. Therefore, we suggest that community inclusion be placed at the center of green infrastructure development. Also, urban foresters, planners, and dendrologists that participate in green infrastructure projects should be trained in inclusive community engagement to secure beneficial outcomes for residents.

In order for communities to be engaged in air pollution mitigation in a meaningful way, they need access to inventories of emissions data and ambient air quality monitoring in formats that are user-friendly and publicly available. This is essential for affected communities, researchers, and other stakeholders to have accurate data for mitigation measures like green infrastructure. Recognizing that air pollution can negatively impact health at multiple scales, mitigation strategies must note the importance of green infrastructure policies at larger (e.g., regional) geographic levels^42^. Likewise, infrastructure to monitor air pollution should be refined at smaller geographic scales, such as census block groups or city blocks, that more accurately reflect community demographics. Current regulatory monitoring does not adequately represent disparate air pollution exposures at the community scale, particularly for low-income and communities of color^43^. Accounting for these challenges relates to quantifying air pollution exposure in different locations^44^ and understanding spatial patterns of air quality.

While we acknowledge the importance of equal access to green infrastructure, it is crucial to ensure that related professionals uphold ethical practices and dismantle further marginalization. Many organizations have standards related to justice and fair treatment of all people in their mission^41,42^, but the actual implementation of these standards in green infrastructure projects needs to be evaluated. A code of ethics is shortsighted without objective oversight in place to verify that it is being practiced. Therefore, we suggest more emphasis on how environmental, urban planning, and public health professionals are evaluated on their ethical practices.

In many sectors of society, marginalized people and communities are treated without the value and significance that they inherently possess. To actualize equitable policies on green infrastructure and air pollution, we must honor the moral and ethical tenets of equal protection under our laws (and create those that do not exist). This means reducing pollution exposure and repositioning vulnerable populations to receive ecosystem services. Although scholars have long expressed how systemic racism and disenfranchisement create health disparities^45,46^, the seeds of inequality planted centuries ago have perpetuated unequal exposure to environmental hazards and access to environmental benefits. Developing green infrastructure projects can bring great benefits to marginalized communities, but cannot on its own solve historical and systemic inequality. In the same way that we invest to protect ecological diversity, we must act to eliminate inequity for marginalized people by effectively partnering nature with all of the people that it supports.
